# Dioxin-Contaminated Farmed Salmon: Foran et al. Respond

**Published:** 2005-10

**Authors:** Jeffery A. Foran, David O. Carpenter, M. Coreen Hamilton, Barbara A. Knuth, Steven J. Schwager

**Affiliations:** Midwest Center for Environmental Science and Public Policy, Milwaukee, Wisconsin, E-mail: Jforan@mcespp.org; Institute for Health and the Environment University at Albany, Rensselaer, New York; AXYS Analytical Services Ltd., Sidney, British Columbia, Canada; Cornell University, Ithaca, New York

Middaugh et al. suggest that relying strictly on risk assessment to develop fish consumption advice has many shortcomings. We agree. They also argue that risk assessment is only part of the risk management process. Although we separate risk assessment and risk management, we agree conceptually that risk management decisions often must be based on more than just the results of a quantitative risk assessment.

What Middaugh et al. fail to recognize is that our report on dioxins in salmon (Foran et al. 2005) was not intended to serve as a fish consumption advisory. Such advisories should be left to appropriate state, federal, and international organizations charged with protection of public health. Rather, we reported risk-based consumption advice that would be triggered by dioxin-like compounds (DLCs) in farmed Atlantic and wild Pacific salmon using two different approaches; the World Health Organization (WHO) tolerable daily intake (TDI) for DLCs and a margin-of-exposure approach advocated by the U.S. Environmental Protection Agency (EPA 2002). We also reported cancer risks, based on the proposed U.S. EPA cancer slope factor for DLCs (U.S. EPA 2002) that would be generated at particular salmon consumption levels. Our results demonstrate clearly that consumption of some farmed Atlantic salmon, even at relatively modest levels, raises human exposure to DLCs above the lower end of the WHO TDI and considerably above background DLC intake for adults in the United States. Further, consumption at these levels poses elevated cancer and noncancer health risks.

Middaugh et al. suggest that human biomonitoring should be used rather than relying on calculated estimates of exposure, presumably to generate fish consumption advice. We strongly disagree, particularly in the case where the exposure source (farmed Atlantic salmon) is not localized. This is a global problem that would require human biomonitoring on immense temporal and spatial scales. In this case, quantitative risk assessment, which includes an assessment of chemical fate, transport, exposure, and effects, is an appropriate surrogate for human biomonitoring. Further, given our vast knowledge of the toxicokinetic behavior and toxicologic effects of dioxin and other bioaccumulative compounds in farmed Atlantic salmon, requiring human biomonitoring before issuing consumption advice is akin to continuing a clinical trial of a drug where unacceptable adverse effects have already been demonstrated. Clearly, responsible public health professionals should strenuously object to such an approach.

Middaugh et al. suggest that two aspects of our study are problematic. First, they argue that measuring contaminants in skin-on fillets may overestimate contaminant concentrations in edible fish tissue and, ultimately, human exposure. We addressed this issue in our article (Foran et al. 2005). We encourage Middaugh et al. to reexamine our conclusion that most studies of the effects of preparation (including removal of skin) and cooking on contaminant concentrations in fish tissue

suffer from small sample sizes, questionable data analyses, inconsistent analytical techniques, inconsistent data presentation, and variability in initial and postintervention contaminant concentrations within and among species, preparation techniques, and cooking techniques. Deficiencies in study design and variability in contaminant reductions preclude development of a useful quantitative correction factor for the effects of preparation and cooking on contaminant burden. As a result, reductions in exposure and risk associated with reduction in contaminant concentrations from preparation and cooking cannot be evaluated quantitatively; thus, we have not incorporated the effects of cooking and preparation in our risk assessments.

Second, Middaugh et al. are correct in stating that we did not adjust for the existing background concentration that incorporates DLC exposure via fish consumption. However, we did assess such exposures and concluded that they were so low, compared with exposure to DLC through consumption of farmed Atlantic salmon, as to be inconsequential in our risk assessment calculations.

Finally, we regret that Middaugh et al. ignored two critically important conclusions of our work. First, in all of our articles (Hites et al. 2004a, 2004b; Foran et al. 2004, 2005) that address contamination of salmon sold commercially, we provided information that will allow and encourage consumers to choose other fish, including wild Pacific salmon, as well as other sources of beneficial n-3 fatty acids. Second, our work has exposed serious deficiencies and inconsistencies in national and international approaches to the management of contaminants in commercially sold fish. These deficiencies and inconsistencies must be resolved so that consumers can confidently choose and consume fish with lower contaminant concentrations while continuing to accrue the health benefits of fish consumption.
